# Training Population Optimization for Prediction of Cassava Brown Streak Disease Resistance in West African Clones

**DOI:** 10.1534/g3.118.200710

**Published:** 2018-10-29

**Authors:** Alfred Ozimati, Robert Kawuki, Williams Esuma, Ismail Siraj Kayondo, Marnin Wolfe, Roberto Lozano, Ismail Rabbi, Peter Kulakow, Jean-Luc Jannink

**Affiliations:** * National Crops Resources Research Institute (NaCRRI), P.O. Box, 7084 Kampala, Uganda; † School of Integrative Plant Science, Plant breeding and Genetics Section, Cornell University, Ithaca, New York; ‡ International Institute for Tropical Agriculture (IITA), Ibadan, Oyo, Nigeria; § United States Department of Agriculture, Agricultural Research Service (USDA-ARS) R.W. Holley Center for Agriculture and Health, Ithaca 14853, NY

**Keywords:** Cassava, genomic selection, training population, and cassava brown streak disease, Genomic Prediction, GenPred, Shared Data Resources

## Abstract

Cassava production in the central, southern and eastern parts of Africa is under threat by cassava brown streak virus (CBSV). Yield losses of up to 100% occur in cases of severe infections of edible roots. Easy illegal movement of planting materials across African countries, and long-range movement of the virus vector (*Bemisia tabaci*) may facilitate spread of CBSV to West Africa. Thus, effort to pre-emptively breed for CBSD resistance in W. Africa is critical. Genomic selection (GS) has become the main approach for cassava breeding, as costs of genotyping per sample have declined. Using phenotypic and genotypic data (genotyping-by-sequencing), followed by imputation to whole genome sequence (WGS) for 922 clones from National Crops Resources Research Institute, Namulonge, Uganda as a training population (TP), we predicted CBSD symptoms for 35 genotyped W. African clones, evaluated in Uganda. The highest prediction accuracy (r = 0.44) was observed for cassava brown streak disease severity scored at three months (CBSD3s) in the W. African clones using WGS-imputed markers. Optimized TPs gave higher prediction accuracies for CBSD3s and CBSD6s than random TPs of the same size. Inclusion of CBSD QTL chromosome markers as kernels, increased prediction accuracies for CBSD3s and CBSD6s. Similarly, WGS imputation of markers increased prediction accuracies for CBSD3s and for cassava brown streak disease root severity (CBSDRs), but not for CBSD6s. Based on these results we recommend TP optimization, inclusion of CBSD QTL markers in genomic prediction models, and the use of high-density (WGS-imputed) markers for CBSD predictions across population.

Cassava (*Manihot esculenta* Crantz) is ranked the fourth most important source of calories in the developing world, after wheat, maize, and rice, and is estimated to feed a population of about 700 million people directly or indirectly (Legg *et al.*, 2014). Reports on global cassava production in the 1960s positioned Brazil as the leading producer in the world, however in the 1990s Nigeria became the world’s largest cassava producer, accounting for half of the world’s total production (Nweke 2004). Other African countries where cassava is a major staple food crop include Uganda, Tanzania and Kenya in eastern Africa, Malawi and Mozambique in southern Africa, Democratic Republic of Congo (DRC) in central Africa, and Ghana in western Africa (Hillocks and Jennings 2003). Cassava is popular in Africa as a food security crop, because of its resilience under drought and poor soils, and its ability to be easily propagated through stem cuttings (Masona *et al.*, 2001; Legg *et al.*, 2014).

Yields of cassava have remained low (8-12 tons/ha) in Africa compared to Asian countries such as Thailand and Vietnam where yield averaged are up to 20 tons/ha (Nweke 2004). Reasons for relatively low yields in Africa include both abiotic (low soil fertility and socio-economic factors such as lack of access to improved varieties) and biotic factors (Nweke 2004). The most devastating biotic stresses today are the cassava brown streak (CBSD) and cassava mosaic (CMD) diseases (Maruthi *et al.*, 2005; Mware *et al.*, 2009). Of these two virus-induced diseases, CBSD is the most important constraint to cassava production in central, eastern and southern Africa as it causes yield losses of up to 100% (Alicai *et al.*, 2007; Hillocks *et al.*, 2016).

Phylogenetic analysis of complete viral RNA genome sequences taken from CBSD symptomatic plants, sampled across eastern and southern Africa, revealed two clades of distinct CBSD-causing virus species that were named: Uganda cassava brown streak virus (UCBSV) and cassava brown streak virus (CBSV) (Winter *et al.*, 2010; Mohammed *et al.*, 2011; Patil *et al.*, 2015; Alicai *et al.*, 2016; Mbewe *et al.*, 2017). The two species belong to genus *Ipomovirus* within the family of *Potyviridae*, and share an identity of 70% and 74% at the level of nucleotide and polyprotein amino acid sequences, respectively (Monger *et al.*, 2001; Winter *et al.*, 2010). Cassava brown streak disease symptoms on cassava leaves manifest as feathery chlorosis around secondary veins, which may disappear when new growth starts after a period of drought-induced leaf abscission (Hillocks 2004). While on the roots, CBSD symptoms externally present as radial constriction, and internally as brown necrotic lesions on part or all of the starchy root, making it inedible (Hillocks 2004; Hillocks *et al.*, 2016).

Although the first incidence of CBSD was reported in 1930s (Storey and Nichols 1938), little attention was paid to it, because geographically CBSD was confined to the low altitudes of east African coastal region (less than 1000 m.a.s.l). Nonetheless, CBSD has spread rapidly to other countries including; Uganda, Burundi, DRC, Mozambique and Rwanda in the last two decades to cover wider range of altitudes than previously reported (Hillocks *et al.*, 2002; Alicai *et al.*, 2007; Legg *et al.*, 2011; Mulimbi *et al.*, 2012). Cassava brown streak disease is commonly spread through sharing of infected stem cuttings for propagation, in addition to super-abundant whitefly *Bemisia tabaci*, as a vector (Hillocks and Jennings 2003; Njoroge *et al.*, 2017).

Officially, genetic materials can move from W. Africa to E. Africa, but movement in the reverse direction is prohibited to prevent accidental introduction of CBSD-causing viruses in W. Africa. Nevertheless, the free movement of planting materials across farming communities has led to increased fear that CBSD could spread to other regions, including West Africa (Legg *et al.*, 2014; Patil *et al.*, 2015; Beyene *et al.*, 2017). Given the current impact of CBSD on cassava production in endemic countries, effort needs to be in place to avert or minimize future CBSD impact in W. Africa, especially Nigeria the world’s leading cassava producer. Among other methods, Legg *et al.*, (2014) proposed pre-emptive breeding for CBSD resistant clones in W. Africa.

High levels of field resistance to CBSD have been reported from genetically transformed plants with coat protein of UCBSV and CBSV, compared to non-transformed plants (Ogwok *et al.*, 2012; Odipio *et al.*, 2014; Beyene *et al.*, 2017; Wagaba *et al.*, 2017). However, the transgenic CBSD resistant clones are still within research confinement, because of unclear regulatory frameworks regarding field production of genetically modified organisms (GMO) in Uganda and east Africa at large. Other efforts to breed for CBSD resistance in E. Africa are geared toward identification of quantitative trait loci (QTL) for CBSD resistance, with the aim of developing molecular markers to implement marker-assisted selection (MAS). A number of QTL mapping studies for CBSD resistance in E. African germplasm have been conducted, and the studies pointed out both unique and overlapping QTL regions for which markers could be developed for MAS (Kayondo *et al.*, 2018; Masumba *et al.*, 2017; Nzuki *et al.*, 2017). One of the highest effect QTL detected involved a bi-parental mapping population from a cross between Kiroba and AR37-80 that explained 18% of total phenotypic variance (Nzuki *et al.*, 2017). However, using bi-parental QTL to develop markers for MAS is only feasible if the QTL are validated in other breeding populations. Furthermore, the recent genome-wide association studies conducted by Kayondo *et al.* (2018), using same training populations, confirmed the polygenic nature of CBSD resistance previously reported (Kawuki *et al.*, 2016).

Genomic selection, proposed by Meuwissen *et al.* (2001) provides an option for using DNA markers for traits that are truly quantitative, where no single causal locus accounts for a major fraction of the variation for selection decisions. Genomic selection (GS) relies on a genome-wide distribution of markers to ensure all QTL have at least one marker in high LD, enabling selection on highly polygenic traits. Genomic selection is typically done using a phenotyped and genotyped training population to estimate genome-wide marker effects (Hayes *et al.*, 2009). The genomic estimated breeding values (GEBV) for all genotyped individuals can then be computed as the sum of marker effects multiplied by the marker genotypes across the whole genome (Meuwissen, *et al.*, 2001). These GEBVs aim to capture all QTL accounting for variation in target traits (Hayes *et al.*, 2009).

Although GS has reportedly outperformed traditional selection methods such MAS and marker assisted recurrent selection (MARS) for quantitative traits (Goiffon 2016), successful implementation of genomic selection depends on a number of factors including: trait heritability, marker density, the size of the training population, the relationship between the training population (TP) and the selection candidates (Jannink *et al.*, 2010; Heffner *et al.*, 2011; Nielsen *et al.*, 2016).

Increases in prediction accuracy have been reported by composing training populations from optimal subsets of individuals chosen to minimize the expected prediction error variance (PEV) of the selection candidates compared to using random subsets or even the full set of available individuals (Rincent *et al.*, 2012; Akdemir *et al.*, 2015; Isidro *et al.*, 2015; Yu *et al.*, 2016). Furthermore, studies have shown increased prediction accuracies with inclusion of prior QTL information in genomic prediction models. For example, a study by Haffliger, (2016) for reproductive traits in Swiss pig breeds revealed a significant increase in prediction accuracy for piglets when previously detected reproductive trait QTL markers were included in the prediction model.

Thus, this study aimed to evaluate the use of genomic predictions of West African clones using training data from a Ugandan population as a pre-emptive breeding strategy for CBSD resistance. Specifically we tested CBSD prediction accuracies for (i) different sizes of training populations across genomic prediction models (ii) random and optimized training sets, (iii) models with incorporation of prior CBSD QTL, and (iv) high and low density marker panels.

## Materials and Methods

### Constitution and evaluation of training population

The training population comprised 922 clones, combined from two experimental trials. For consistency, we refer to the trials as training population 1 (TP1) and training population 2 (TP2). A total of 400 clones (TP1) were generated from crossing diverse parents that were assembled from International Center for Tropical Agriculture (CIAT), International Institute of Tropical Agriculture (IITA), Tanzania, and National Crops Resources Research Institute (NaCRRI), Uganda. The introductions from CIAT targeted improvement for quality and yield traits, while the germplasm from the IITA, Tanzania and NaCRRI breeding programs targeted resistance to CBSD.

Crosses were made among the progenitors to generate TP1 in 2009-2010, from which both controlled crosses and open-pollinated seeds were harvested. After seedling evaluation, the first clonal evaluation for TP1 was done at Namulonge in 2012-2013 by conducting an un-replicated experiment, and afterward expanded to three sites (Kasese, Ngetta and Namuloge) for the second year of clonal evaluation, planted in alpha lattice design, in single row plots of 10 plants, replicated twice.

The second training population (TP2) comprised 522 clones, generated from open-pollinated seeds that were harvested from the first clonal evaluation trial of TP1. Similar to TP1, after a year of seedling evaluation (2013-2014), TP2 was planted for the first clonal evaluations in 2014 at two sites (Namulonge and Kamuli). In 2015, TP2 was replanted for the second year of clonal evaluation, with the trials expanded to three sites (Namulonge, Kamuli, Serere). Thus, Namulonge was the only overlapping evaluation site between TP1 and TP2. The clonal evaluations for TP2 were established in an augmented incomplete block design with six common checks per block, and each plot within a block containing 10 plants established in a single row. Planting of all the trials was done at spacing of 1 m × 1 m adopted within and between rows, while blocks were separated by 2 m alleys.

Data on foliar CBSD severity was collected at three and six months after planting (MAP), while the roots were evaluated for CBSD severity at 12 MAP. Foliar severity for CBSD was assessed on a scale of 1-5 (Hillocks and Thresh 2000), where: 1 = no symptom; 2 = slight foliar chlorotic leaf mottle with no stem lesions; 3 = foliar chlorotic leaf mottle and blotches with mild stem lesions, but no die back; 4 = foliar chlorotic leaf mottle and blotches with pronounced stem lesions, but no die back; and 5 = defoliation with stem lesions and dieback. To assess root necrosis severity, each root was sliced transversely 5-7 times and the cross-sections scored for necrotic symptoms on a scale of 1-5 (Hillocks and Thresh 2000), where: 1 = no necrosis, 2 = ≤ 5% necrotic; 3 = 6–10% necrotic; 4 = 11–25% necrotic and mild root constriction; and 5 = >25% necrotic and severe root constriction.

### West African genetic materials and evaluation

In 2015, we received a total of 95 clones that constituted part of IITA, Nigeria genetic gain population for implementing genomic selection (Wolfe *et al.*, 2017). These clones were shipped to Uganda in the form of tissue culture plantlets. The first set of 30 clones was received in February 2015 and the second lot of 65 clones was received in June 2015. The plantlets were multiplied in tissue culture and further hardened in a screen house for three months.

In August and November 2015, the first set of 30 and the second set of 65 clones were planted in the field at Namulonge. This was done to generate adequate stem cuttings for establishment of replicated field trials. In September 2016, we established a trial for the first set of 27 clones that survived, in a randomized complete block design (RCBD) replicated twice, with each plot containing 10 plants in a single row.

For the second set of 65 clones, unfortunately we lost more than half of the clones due to drought that occurred a month after their first field exposure in 2015. The remaining 22 clones that survived were planted in November 2016, again using an RCBD, replicated twice. In contrast to the first set of 27 clones, there was only enough planting material for five plants per plot. Cassava brown streak disease phenotyping was conducted as described previously. All infections occurred under natural conditions.

### DNA extraction and genotyping

Approximately 100 mg of fresh tissue was collected from tender apical leaves of TP1 and TP2 clones for DNA extraction. DNA was extracted following the protocol for the QIAGEN DNeasy extraction kit and quantified using the PicoGreen DNA quantification kit to ensure the required concentrations were obtained for sequencing. The extracted DNA samples were shipped to the Cornell University Genomics Diversity Facility for genotyping, using the genotyping-by-sequencing (GBS) approach (Elshire *et al.*, 2011). The GBS libraries were constructed using the ApeKI restriction enzyme as described previously (Rabbi *et al.*, 2014).

Marker genotypes were called using TASSEL GBS pipeline v4 (Glaubitz *et al.*, 2014), after aligning the reads to the Cassava reference genome v6 (Prochnik *et al.*, 2012). Using VCFtools, Variant Calling Format (VCF) files were generated for each chromosome. Genotypes with less than five reads were masked before imputation. Similarly, markers with more than 60% missing calls were removed. Only bi-allelic GBS SNP markers were considered for further processing. Missing markers were imputed using Beagle 4.1 software (Browning and Browning 2016), with default parameter settings. In all, 46,760 SNPs remained, which we referred to as the “GBS” markers.

In addition, we used a second set of markers, which we referred to as “whole genome sequence” imputed (or “WGS-imputed”) markers. Using IMPUTE2 software, the GBS samples were imputed to a marker set equivalent to what we would get from actual whole genome sequencing. The details of the imputation procedure are described by Lozano *et al.*, (2017). Briefly, the WGS imputation relied on the cassava HapMapII, a collection of 241 genome-sequenced samples (Ramu *et al.*, 2017) as a reference panel. This reference panel comprised mainly of improved cassava clones under cultivation and a few wild relatives, and contained ∼28 million SNP markers (Lozano *et al.*, 2017). The set of markers referred to as “WGS-imputed” hereafter, included ∼5 million SNPs, after filtering for minor allele frequencies (MAF) ≥ 0.01.

### Statistical analyses

#### Analyses of phenotypic data:

Because of differences in trial design for TP1 and TP2 as well as the IITA clones, two-step genomic prediction analyses were done. In the first step of the analyses, linear mixed models accounting for each trial’s design were fitted and de-regressed BLUPs were obtained for TP1 and TP2. For TP1, we fitted the model:$\;y=X\beta +Z_{clone}c+Z_{range(loc.)}r+Z_{block(range)}b+\varepsilon$, using the *lmer* function from the *lme4* R package (R Development Core Team 2008). In this model, ***β*** defined the fixed effect for the population mean and location, with X as the corresponding incidence matrix. The incidence matrix **Z_clone_** and the vector *c* represented random effect for clones $\;c∼\text{N}\left(0,I\sigma_{c}^{2}\right)$, and I represented the identity matrix. The range variable, which was the row or column along which plots were arrayed, was nested in location-replication and was represented by the incidence matrix **Z_range(loc.)_** and random effects vector $r∼\text{N}\left(0,I\sigma_{r}^{2}\right)$. Block effects were nested in ranges and incorporated as random term with incidence matrix **Z_block(range)_** and effects vector $b∼\text{N}\left(0,I\sigma_{b}^{2}\right)$. Residuals ε were distributed as$\;\varepsilon ∼\text{N}\left(0,I\sigma_{\varepsilon }^{2}\right)$.

For TP2 (522 clones), we fitted the linear mixed model $\;y=X\beta +Z_{clone}c+Z_{block}b+\varepsilon$, where *y* was the vector of raw phenotypes, ***β*** included a fixed effect for the population mean and location with checks included as a covariate. The incidence matrix **Z_clone_** and the vector *c* were similar for both TP1 and TP2. The blocks were also modeled with incidence matrix$\;Z_{block}$, and ***b*** represented the random effect for the blocks. The best linear unbiased predictors (BLUPs) of the clone effects were extracted as de-regressed BLUPs following the formula proposed by Garrick *et al.*, (2009).

$$\text{deregressed BLUP}=\frac{\text{BLUP}}{1-\frac{\text{PEV}}{\sigma_{\text{c}}^{2}}}$$

Here, PEV represented the prediction error variances for the BLUPs and $\sigma_{\text{c}}^{2}$ was the clone variance.

For the IITA clones, we fitted the following mixed model:$\;y=X\beta +Z_{clone}c+Z_{rep(trial)}b+\varepsilon$. Where *y* was a vector of raw phenotypes, ***β*** included a fixed effect for the population mean. The incidence matrix **Z_clone_** and the vector *c* represented random effect for clones $c∼\text{N}\left(0,I\sigma_{c}^{2}\right)$ and I represented the identity matrix, and the replication nested in the trials was modeled with incidence matrix$\;\;Z_{rep(trial)}$, with random effect ***b*** representing replications nested within trial for the first set of 27 and second set of 22 clones, with 14 overlapping clones between the sets. The best linear unbiased predictors (BLUPs) were extracted from the model and subsequently used as the validation data for estimation of genomic prediction accuracies of CBSD for the 35 unique IITA clones. In addition, variance components were extracted from the model to compute plot based broad-sense heritability estimates.

#### Population structure:

To assess population structure, we used the GBS markers of TP1, TP2, and the 35 IITA clones. These markers were filtered to have MAF ≥ 0.01 and formatted as a dosage matrix with SNP genotypes coded as -1, 0, or +1. Principal component analysis (PCA) was done on the SNP matrix, using the *prcomp* function in R. The first two principal components (PC) were used to visualize population structure.

#### Cross-validation prediction accuracies for IITA clones:

Weestimated prediction accuracies for foliar CBSD severities evaluated at three (CBSD3s) and six (CBSD6s) months, and root severity at 12 months (CBSDRs), using a fivefold cross validation scheme, replicated 10 times for IITA clones from a single-step genomic best linear unbiased predictor (G-BLUP) model. For each replication in the cross-validation scheme, the 35 IITA clones were randomly divided into five groups of 7 clones each (folds). Four groups at a time were used as the training population to build the prediction model, while excluding the fifth group, which was used as the model validation set. This was repeated for all the 5-folds for each of the 10 replications. Prediction accuracies were computed as the Pearson correlation coefficient between the genomic estimated breeding values predicted for the validation set and the corresponding BLUPs obtained from the first-step of the analysis for 35 unique individuals in the test set (IITA clones).

#### Genomic prediction of CBSD for IITA clones:

We tested genomic prediction accuracies under four scenarios: (i) optimized training populations across genomic selection models (ii) optimized *vs.* random subset training populations for G-BLUP only (iii) models with inclusion of kernels defined by chromosomes on which CBSD QTL have been found (single and multi-kernel G-BLUP models), and (iv) high and low density marker panels for G-BLUP model.

To optimize the training population, we used the selection of training population with a genetic algorithm (STPGA), *GenAlgForSubsetSelection*, from the R package STPGA (Akdemir *et al.*, 2015). The algorithm identifies a subset of a specified size from a larger pool of potential training individuals. To do this, STPGA finds the set of individuals that minimize the mean prediction error variance (mean PEV) expected for test set, using molecular marker data.

For STPGA training population optimization, we used the first 50 principal components (PC’s) of the eigenvalue decomposition of the marker matrix as a dimension reduction approach. The pool of potential training individuals was the combined TP1 and TP2 (N = 922) described above and the target or test set were the IITA clones. We optimized 20 training populations within each size of training population specified in STPGA. In scenario (i), the optimum training populations for each training population size (100, 200, 400, 800 and full set = 922) were used to predict CBSD with four genomic prediction models namely; G-BLUP, Bayes-A, Bayes-B and Bayesian Lasso (Lorenz *et al.*, 2011; Heslot *et al.*, 2012).

Under scenario (ii), we tested the performance of STPGA by comparing the optimized sets from scenario (i) with random subsets of the same size. We chose to compare optimized and random sets for population of sizes of 200 and 400, based on results from analyses in scenario (i), and for each training size we compared 20 sets for both random and optimized TPs, using G-BLUP model because of it robustness and computational efficiency.

In the single kernel model, all GBS markers were fitted with one realized genomic relationship matrix K, according to the formula described by VanRaden, (2008). The relationship matrix was constructed using *A.mat* function in rrBLUP package (Endelman 2011). The model was specified as: $y=1_{n}u_{0}+Zg+e,\text{ with}$ $g∼N(0,K\sigma_{g}^{2})$ and $\;e∼N(0,I\sigma_{e}^{2})$, where y was the vector of de-regressed BLUPs, $u_{0}$ was an overall population mean, Z was the design matrix linking observations to genomic values, g was the vector of genomic estimated breeding values for each clone, and e was the vector of residuals. We assumed, *g* had a known covariance structure defined by the realized genomic relationship matrix *K*.

Previously, QTL for CBSD have been reported on chromosomes 4 and 11 (Kawuki *et al.*, 2016; Kayondo *et al.*, 2018). We used all the markers on the two chromosomes (Chr.4 and 11) because significant markers covered essentially the whole of Chr. 4 for CBSD6s and about half of Chr. 11 for both CBSD3s and CBSD6s (Kayondo *et al.*, 2018). Therefore, we also fitted a multi-kernel G-BLUP model with two realized genomic relationship matrices, constructed using *A.mat* function as described above. In this model, the first genomic relationship matrix incorporated all markers from both chromosome 4 and 11, while the second genomic relationship matrix was derived from the rest of the genomic markers. The model was: y = $1_{n}u_{0}+Zq+Zr+e$. Here, y was the vector of de-regressed BLUPs, $u_{0}$ was an overall mean, *Z* was the design matrix linking observations to genomic values, *q* was the vector of genomic values captured by combined QTL markers linked to CBSD resistance, *r* was the vector of genomic values captured by the remaining set of genetic markers, and e was a vector of residuals. The random genetic effects for both kernels with their variance-covariance structure *K*, and the residuals were assumed to be normally distributed as $q∼N(0,K_{q}\sigma_{q}^{2})$, $r∼N(0,K_{r}\sigma_{r}^{2})$ and $e∼N(0,I\sigma_{e}^{2})$.

Furthermore, we fitted a multi-kernel G-BLUP model with three genomic relationship matrices, where the first and second realized genomic relationship matrices were defined by all the markers on chromosomes 4 and 11 respectively, while the third kernel contained markers from the remaining 16 chromosomes. The model was: y = $1_{n}u_{0}+Zp+Zs+Zr+e$. Here, y was the vector of de-regressed BLUPs, $u_{0}$ was an overall mean, *Z* was the design matrix linking observations to genomic values, p and s were the vectors of genomic values captured by QTL markers on chromosome 4 and 11 respectively, *r* was the vector of genomic values captured by the remaining set of genetic markers, and e was the vector of residuals. The random effects, including the residual-term were assumed to be normally distributed as $\;p∼N(0,K_{p}\sigma_{p}^{2})$, $∼N(0,K_{s}\sigma_{s}^{2})$,$\;r∼N(0,K_{r}\sigma_{r}^{2})$ and $e∼N(0,I\sigma_{e}^{2})$.

For both single and multi-kernel G-BLUP analyses, we used the two EMMREML functions, *emmreml* and *emmremlMultiKernel* to fit single and multi-kernel G-BLUP models respectively (Akdemir and Okeke 2015). Lastly, we tested prediction accuracies of CBSD3s, CBSD6s and CBSDRs using high-density (WGS-imputed) markers and compared that to low-density (GBS) markers used in the analyses described above. For the high-density set, we fitted the single- and multi-kernel G-BLUP models described above, using training populations of 200 and 400 clones that were optimized using either the GBS or the WGS markers. Because the results of these two optimizations were quite similar, we only reported results from the GBS optimizations.

### Data availability

All the raw phenotypic and genotypic data are available at the link provided for references. Supplemental material available at Figshare: https://doi.org/10.25387/g3.7242851.

## Results

### Population structure, heritability and cross-validation within IITA clones

**Figure 1:**
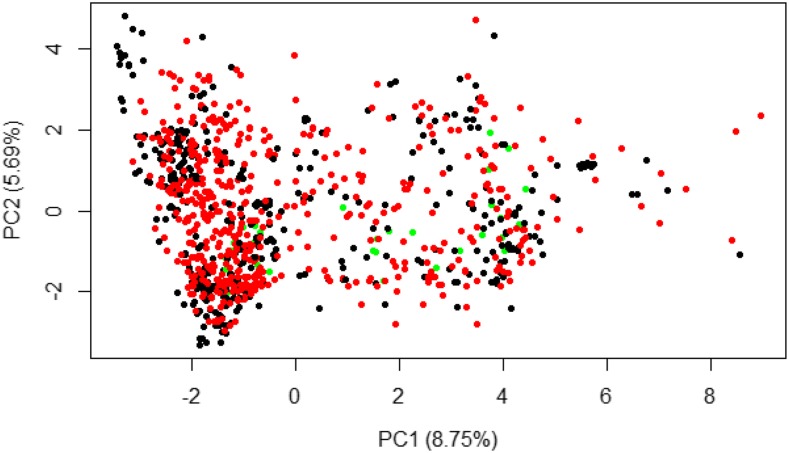
Plot of PC1 against PC2 for Eigen value decomposition of GBS markers for IITA (green), NaCRRI-TP1 (black) and NaCRRI-TP2 (red) clone.

Principal component analyses on the SNP marker matrix showed no genetic differentiation among the TP1, TP2 and IITA clones. This was supported by PC1 and PC2 explaining only 8.75% and 5.69% of the total genetic variations, respectively (Figure 1 and Figure S6). Estimates of plot-basis broad-sense heritability (H^2^) were computed for CBSD3s, CBSD6s and CBSDRs for the 35 IITA clones (Table 1). Broad-sense heritability estimates spanned from 0.42 to 0.64 for CBSD3s and CBSDRs respectively. In addition to broad-sense heritability, we estimated narrow-sense heritability for IITA clones using a single step G-BLUP model.

**Table 1:** Variance component and plot-basis heritability estimates for IITA clones

Source of Variation	CBSD3s	CBSD6s	CBSDRs
Clones	0.13	0.29	1.01
Reps/trial	0.01	0.00	0.00
Residuals	0.31	0.21	0.56
H^2^	0.42	0.58	0.64

CBSD3s = Cassava brown streak disease severity scored at three months, CBSD6s = Cassava brown streak disease severity scored at six months, CBSDRs = Cassava brown streak disease root severity scored at 12 months, H^2^ = plot-based broad-sense heritability estimates.

**Figure 2:**
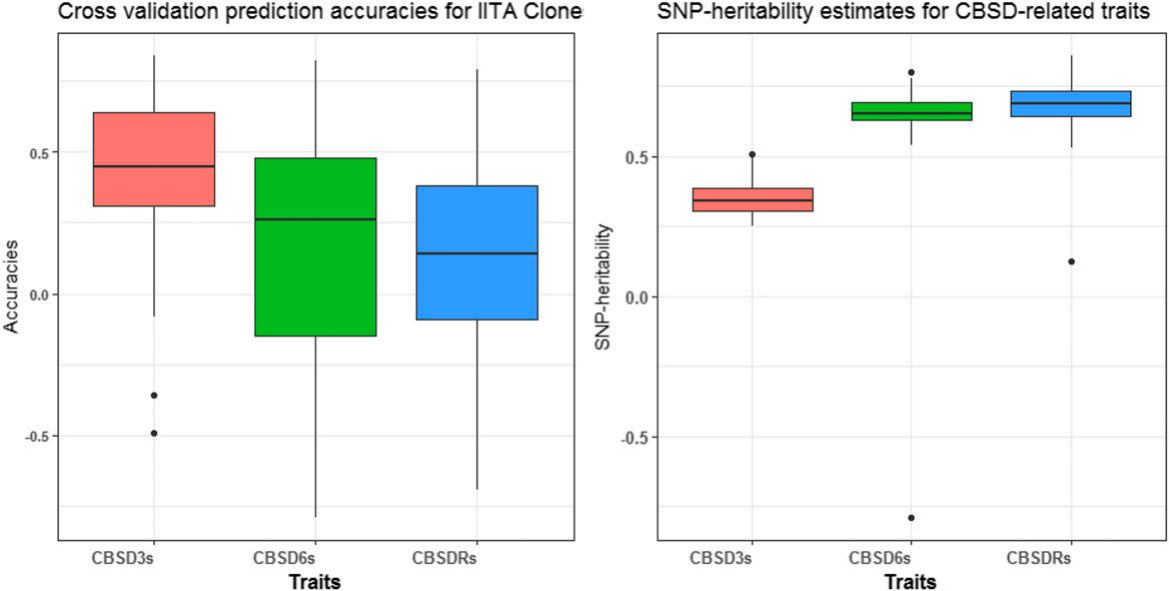
Prediction accuracies for fivefold and 10 reps using G-BLUP model, and SNP-heritability estimates for CBSD3s, CBSD6s and CBSDRs in 35 IITA clones.

The lowest and highest narrow-sense heritability of 0.35 and 0.69 were recorded for CBSD3s and CBSDRs, respectively (Figure 2). The average prediction accuracies from fivefold cross-validation replicated 10 times for the IITA clones were 0.40, 0.21 and 0.08 for CBSD3s, CBSD6s and CBSDRs, respectively (Figure 2). We did not do cross validation within the training set here, because the training population was previously cross-validated (Kayondo *et al.*, 2018). Previous predictive accuracy for CBSD-related traits, had mean values across methods of 0.29 (CBSD3s), 0.40 (CBSD6s) and 0.34 (CBSDRs) for cross-validation within NaCRRI training set.

### Predicting CBSD in IITA clones using Ugandan training population

In general, the mean CBSD prediction accuracies were higher for foliar than root necrosis for the different optimized training population sizes across genomic prediction models (Table 2). For CBSD3s, the prediction accuracies ranged from 0.24 (Bayes-A) to 0.36 (Bayesian Lasso). Prediction accuracies spanned from 0.14 (Bayesian Lasso) to 0.28 (G-BLUP) for CBSD6s. For CBSDRs, accuracies included negative values, ranging from -0.29 (Bayes-A) to 0.11 (Bayes-A) across different optimized training sets.

**Table 2:** Average prediction accuracies (r) for four optimized subsets of TPs and full set across genomic prediction models

Training Size (TP)	G-BLUP			Bayes-A			Bayes-B			Bayesian		Lasso
	CBSD3s	CBSD6s	CBSDRs	CBSD3s	CBSD6s	CBSDRs	CBSD3s	CBSD6s	CBSDRs	CBSD3s	CBSD6s	CBSDRs
TP100	0.27^ns^	0.23^ns^	−0.10^ns^	0.26^ns^	0.22^ns^	−0.19^ns^	0.30*	0.23^ns^	−0.03^ns^	0.33*	0.19^ns^	−0.07^ns^
TP200	0.27^ns^	0.28^ns^	−0.03^ns^	0.26^ns^	0.26^ns^	−0.29^ns^	0.27^ns^	0.26^ns^	0.07^ns^	0.34*	0.22^ns^	0.06^ns^
TP400	0.32*	0.19^ns^	−0.01^ns^	0.32*	0.18^ns^	−0.19^ns^	0.32*	0.17^ns^	−0.09^ns^	0.36*	0.14^ns^	−0.08^ns^
TP800	0.31*	0.26^ns^	0.06^ns^	0.29^ns^	0.25^ns^	−0.13^ns^	0.29^ns^	0.23^ns^	−0.04^ns^	0.31*	0.17^ns^	−0.01^ns^
TP922	0.30*	0.25^ns^	0.05^ns^	0.24^ns^	0.21^ns^	0.11^ns^	0.30*	0.26^ns^	−0.09^ns^	0.31*	0.15^ns^	−0.04^ns^

CBSD3s = Cassava brown streak disease severity scored at three months, CBSD6s = Cassava brown streak disease severity scored at six months, CBSDRs = Cassava brown streak disease root severity scored at 12 months; TP100, TP200, TP400, TP800 and TP922 = Optimized training populations of size 100, 200, 400, 800 and a full set of 922 clones, ns = non-significant prediction accuracies (r), * accuracy significantly different from zero (*P* ≤ 0.05).

The models did not differ much in terms of their prediction accuracies for three traits (CBSD3s, CBSD6s and CBSDRs) across optimized training populations of 100, 200, 400, 800, and full set of 922 clones. Surprisingly, Bayesian Lasso consistently had higher prediction accuracies than the other three prediction models (G-BLUP, Bayes-A and Bayes-B) for CBSD3s across the optimized TP sizes, but performed worse than those three models for CBSD6s across optimized TPs (Table 2).

Prediction accuracies for optimized training populations across the four models tested increased from 100 to 400 for CBSD3s to attain a plateau and declined as the optimized training population was increased to 800 and the full set of 922 clones. However, no clear trend in prediction accuracies were observed for CBSDRs for the different sizes of optimized training population (Table 2).

**Figure 3:**
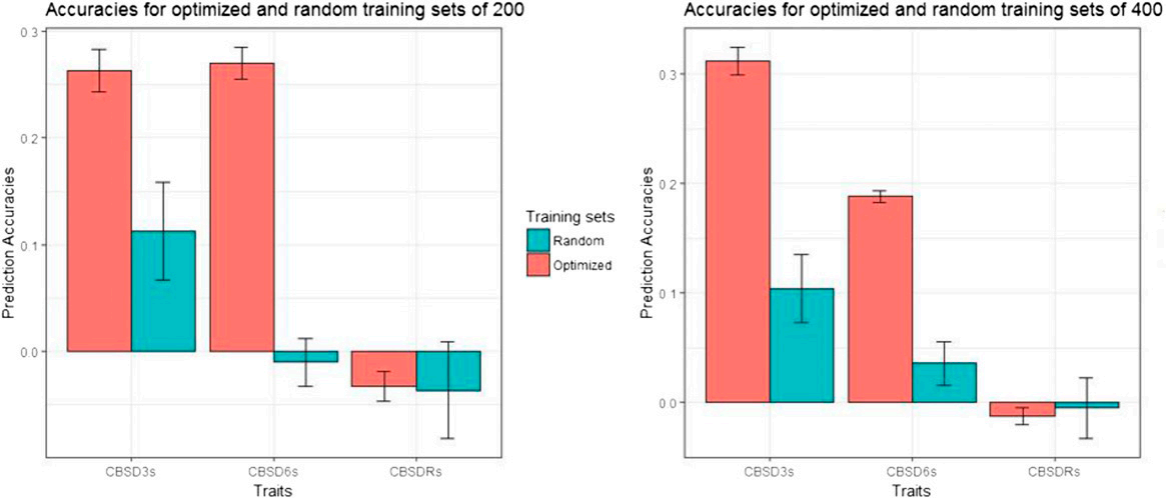
Prediction accuracies and the standard error bars for 20 replications of optimized and random training population size of 200 and 400.

We compared CBSD prediction accuracies from random and optimized training populations of size 200 and 400 clones using the G-BLUP model. We chose these two sample sizes because they maximized prediction accuracies for CBSD3s and CBSD6s (Table 2). For both 200 and 400 clones, the prediction accuracies were higher for optimized training sets than for the random subsets for CBSD3s and CBSD6s (Figure 3; Table S6). For example, at training population size of 200, the mean prediction accuracies for CBSD3s and CBSD6s were 0.27 and 0.28 compared to 0.11 and -0.01 for the corresponding random subsets. Similarly, at training population size of 400 clones, the mean prediction accuracies for CBSD3s and CBSD6s were 0.32 and 0.19 relative to 0.10 and 0.04 for the random subsets (Figure 3; Table S7). We observed markedly lower standard errors as measures of variation in prediction accuracies across the traits for the optimized training populations, compared to the random subsets (Figure 3). However, no strong differences were observed for CBSDRs (Figure 3).

### Accounting for CBSD QTL with chromosome-specific effects or kernels

**Figure 4:**
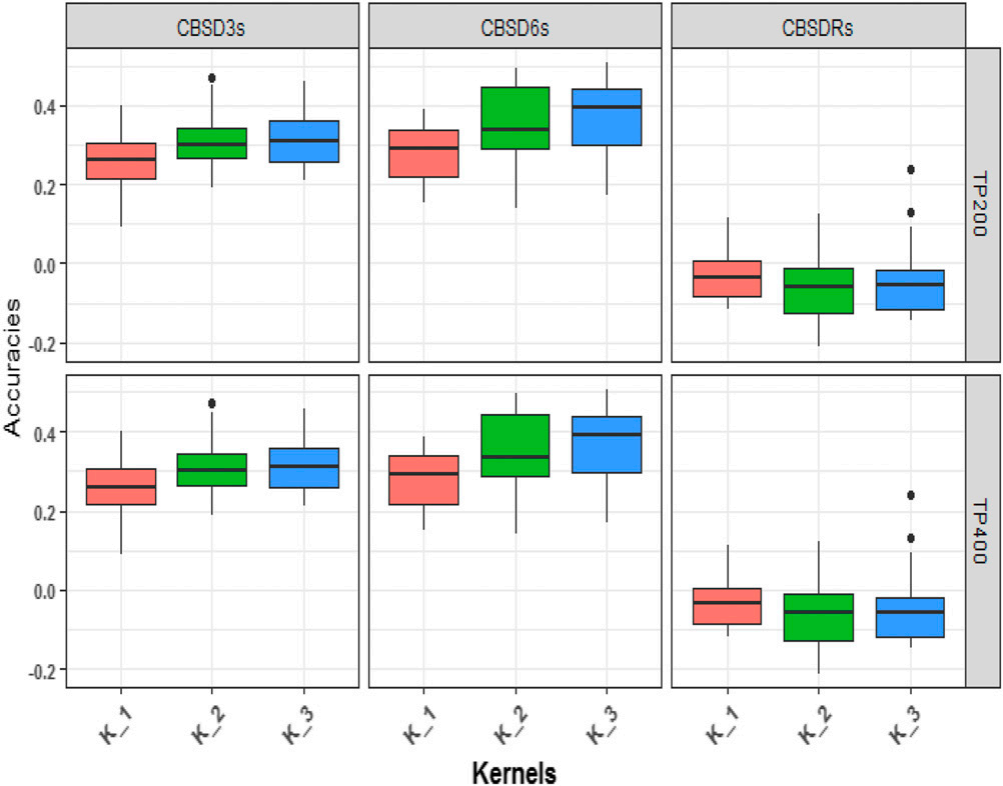
G-BLUP model to compare prediction accuracies for varying number of kernels for CBSD measured at 3, 6 and 12 MAP for size of TP 400 and 200. K_1= Single kernel G-BLUP model. K_2= Multi-kernel G-BLUP, the first kernel is defined by combined markers from chromosomes 4 and 11. The second kernel is defined by the remaining markers. K_3= Multi-kernel G-BLUP, the first and second kernels are defined by markers from chromosomes 4 and 11 respectively, and third kernel is defined by the remaining markers.

In general, foliar CBSD prediction accuracies for training population size of 200 and 400 were higher for multi-kernel models (K_2 and K_3) with separate kernels fitted for CBSD QTL chromosome markers than single kernel (K_1) G-BLUP models (Figure 4). Prediction accuracies for CBSD3s increased from 0.27 for the single kernel G-BLUP, termed as “K_1” model to 0.31 for two-kernel G-BLUP model referred to as “K_2”, and to 0.32 for the three-kernel model referred to as “K_3” in the optimized TPs of 200 clones (Figure 4; Table S8). Similarly, for CBSD6s, prediction accuracies increased from 0.28 for single kernel G-BLUP model to 0.37 with three-kernels (Figure 4; Table S8). No such increase was observed for CBSDRs. Notably, the mean prediction accuracies for CBSD3s and CBSD6s from multi-kernel G-BLUP models were statistically significantly different (*P* ≤ 0.05) from zero. Nevertheless, no differences were observed for CBSDRs prediction accuracies between single- and multi-kernel G-BLUP models at TP size of 200.

For the optimized training population size of 400, a similar trend of increased prediction accuracies was observed from single- to multi-kernel G-BLUP models for both CBSD3s and CBSD6s. The mean prediction accuracies for CBSD6s were not significantly different from zero. Prediction accuracies did not vary much for CBSDRs between single- and multi-kernel G-BLUP models (Figure 4; Table S9).

### Comparing prediction accuracies for high (WGS-imputed) and low (GBS) density markers

**Figure 5:**
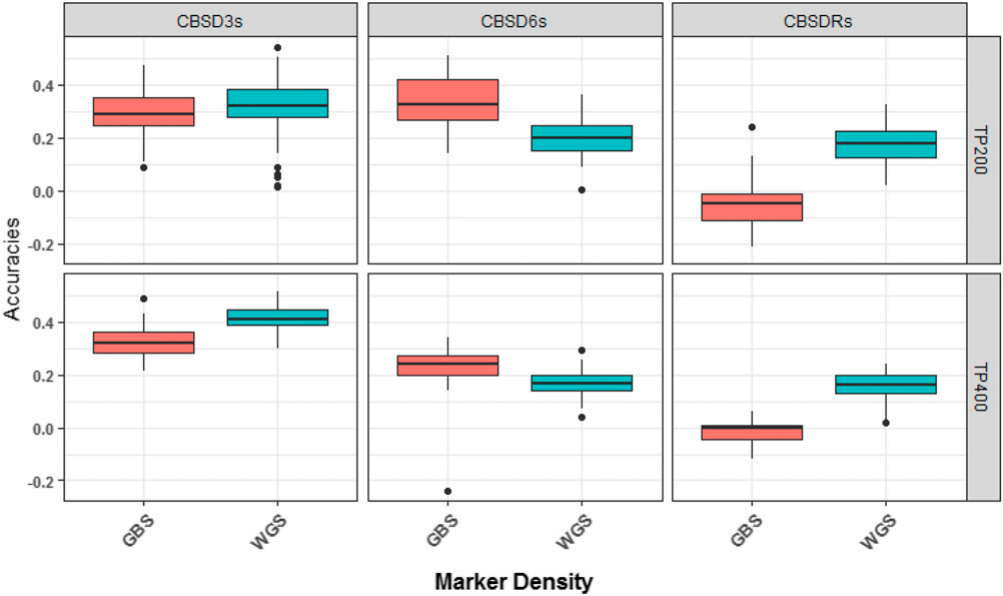
Comparison of prediction accuracies for the CBSD-related traits under high density, whole genome sequence imputed (WGS-imputed) and low density genotyping-by-sequencing (GBS) markers for optimized training population sizes of 200 and 400 clones using single kernel G-BLUP model.

Single kernel G-BLUP prediction accuracies for CBSD3s and CBSDRs were higher for WGS-imputed, than GBS markers for both optimized training population sizes of 200 and 400 clones (Figure 5). For CBSD6s, however, predictions accuracies were lower for high-density (WGS-imputed) at both training population sizes. For single kernel G-BLUP, prediction accuracies for CBSD3s and CBSDRs increased from 0.27 to 0.35, and -0.03 to 0.18 from low to high-density marker sets at the optimized training population size of 200 clones (Table S11). Similarly, predictions accuracies for CBSD3s and CBSDRs increased from 0.32 to 0.39, and -0.01 to 0.16 from low-density (GBS) and high-density (WGS-imputed) markers for the training populations of 400 clones (Figure 5; Table S12).

Fitting multi-kernel G-BLUP models including kernels defined by markers on CBSD QTL chromosomes 4 and 11 for high-density markers did not always increase prediction accuracies (Tables S11 and S12). The highest prediction accuracy of 0.44 (CBSD3s) was recorded from the multi-kernel G-BLUP model, fitted with high density (WGS-imputed) markers for the optimized training population of 400 clones (Table S12). In other cases, prediction accuracies actually dropped from single- to multi-kernel models. For example, prediction accuracy for CBSD3s dropped from 0.35 for single-kernel to 0.32 for multi-kernel (three kernels) at the training population size of 200 clones (Table S11). Overall, the prediction accuracies for high and low-density marker sets were similar between the multi-kernel models regardless of the optimized training population size (Tables S11 and S12).

## Discussion

Cassava brown streak disease (CBSD) caused by Uganda cassava brown streak virus (UCBSV) and cassava brown streak virus (CBSV) has continued to be a major threat to cassava productivity in southern, eastern and central parts of Africa. Recently, CBSD causing viruses were declared the leading biological enemy to cassava productions in CBSD endemic zones of Sub-Sahara Africa (Legg *et al.*, 2014). Concerted efforts such as quarantine, disease surveillance, and breeding for resistance have taken center-stage to prevent further spread of CBSD to W. Africa, especially Nigeria, the world’s largest producer and consumer of cassava. In this paper, we leveraged genome-wide prediction approaches as a potential means to enable pre-emptive breeding for CBSD resistance in W. Africa.

### Impact of different sizes of optimized training population across models

For optimized training populations of 100, 200, 400, 800 and 922 clones, the highest prediction accuracies were observed at the training population sizes of 200 (G-BLUP) and 400 (Bayes-B) clones for CBSD6s and CBSD3s respectively. Our findings were similar to that of Wolfe *et al.* (2017), where prediction accuracy of 0.37 for CMD was observed for both the smallest and largest optimized training sizes of 300 and 900, respectively in cross-population prediction, suggesting that accuracies similar to that of the full set can be obtained with a small but carefully selected TP in relation to the test set.

Overall, the cross-population prediction accuracies for IITA clones, based on optimized training populations and various prediction models (spanning 0.24 to 0.36), were comparable for CBSD3s to those reported previously for the cross-validation within NaCRRI training set, ranging from 0.27 to 0.32 (Kayondo *et al.*, 2018). In contrast, for CBSD6s, our prediction accuracies (0.14 to 0.28) were lower than accuracies reported by Kayondo *et al.* (2018), which ranged from 0.40 to 0.42. The similarity in foliar CBSD prediction accuracy for CBSD3s indicates some genetic signal for CBSD foliar symptom expression for IITA clones was captured by optimal NaCRRI training subsets. Unfortunately, our cross-population prediction accuracies for CBSDRs for optimized TPs were generally lower than the accuracies reported for cross-validation within NaCRRI training population for CBSDRs. In part, the negative prediction accuracies for CBSDR could be explained by GxE interaction for TP1 and TP2 (Table S13 and Figure S4). However, the GxE variances relative to genetic variances were low and therefore unlikely to explain fully the poor prediction accuracies observed for CBSD root necrosis in W. African clones. Also, we observed stronger linkage disequilibrium (LD) in NaCRRI training set than in the IITA test population (Figure S7), suggesting some differences in the LD decay rate between the two populations, which could partly explain the low prediction accuracies observed for CBSDRs in W. African clones. In such a case, there is need to first phenotype clones of W. African descent (*i.e.*, belonging to the W. African subpopulation) in E. Africa, and subsequently use the data generated for predicting CBSD resistance in W. African clones. One option would be to send many W. African clones to E. Africa as tissue culture plantlets. As observed in this study, cost and mortality are high for this option. Another possibility would be to send botanical seeds of W. African clones to E. Africa for evaluation.

We did not observe consistent superior performance for any of the prediction models that we tested or for any of the CBSD traits analyzed. Several studies have reported similar results in that most prediction models perform similarly (Jannink *et al.*, 2010; Heslot *et al.*, 2012; Roorkiwal *et al.*, 2016). Even though the models tested in the present study assumed different distributions of marker effects (Meuwissen *et al.*, 2001; Lorenz *et al.*, 2011), their similarity in prediction accuracies could be interpreted as approximation to optimal genomic prediction models, where all the models capture the same or similar QTL effects across the genome (Su *et al.*, 2014). In such a situation, the choice of GS model would be less important than the actual design of the training population for across-population predictions.

### Comparison of prediction accuracies for random and optimized training populations

Prediction accuracies can be improved by targeting more informative individuals in the reference panel used to generate the predictions and this has been demonstrated in several crop species (Rincent *et al.*, 2012; Akdemir *et al.*, 2015). In general, we observed higher prediction accuracies for CBSD3s and CBSD6s from optimized compared with randomly selected training set of the same size. For example, at TP size of 200 clones, our prediction accuracies for CBSD3s was 0.27 with the optimized compared to 0.11 from the random subset. Similar findings were made by Wolfe *et al.* (2017), where STPGA-optimized training populations performed better than random subsets for a number of important cassava traits, including dry matter content (DMC), harvest index (HI), mean cassava mosaic disease and plant vigor. Our results, therefore, serve to further stress the importance of training population optimization for cross-population prediction.

### Weighting prior biological information for CBSD prediction across population

Studies have shown increased prediction accuracies with inclusion of prior QTL information in genomic prediction models. For example, a study by Haffliger, (2016) for reproductive traits in Swiss pig breeds revealed a significant increase in prediction accuracy for piglets when previously detected reproductive trait QTL markers were included in the prediction model. From the training population used in this study, two recent studies identified CBSD QTL on chromosomes 4 for CBSD3s and CBSD6s, and 11 for CBSD6s and CBSDRs (Kawuki *et al.*, 2016; Kayondo *et al.*, 2018). In addition, bi-parental mapping studies have had similar results (Masumba *et al.*, 2017; Nzuki *et al.*, 2017). In an attempt to improve across-population prediction accuracies for CBSD symptoms, we chose to directly model the Chromosome 4 and 11 (Chr.4 and Chr.11) QTL by incorporating random effects for the markers on those chromosomes into our prediction. Prediction accuracies increased for CBSD3s and CBSD6s, but not for CBSDRs. The benefit was greatest for prediction accuracy of CBSD6s which increased by 9%, when three realized relationship matrices (Chr. 4 + Chr. 11 + the rest, optimized set of 200) were modeled. Although the percentage increase in prediction accuracies was less for the optimized TPs of 400 clones, we still observed increased prediction accuracies for CBSD6s, again when three relationship matrices were fitted. We observed a much higher increase in prediction accuracies for G-BLUP models that included the CBSD QTL as separate random effects, compared to the only marginal increase in prediction accuracies of 1.7% for CBSD3s and 2.5% for CBSDRs reported previously (Lozano *et al.*, 2017).

The higher prediction accuracies we observed by accounting for the CBSD QTL suggests that the development of genomic resources for cassava (Prochnik *et al.*, 2012), the identification of QTL by GWAS (Wolfe *et al.*, 2016; Lozano *et al.*, 2017; Kayondo *et al.*, 2018) and candidate genes by bioinformatics (Lozano *et al.*, 2015) can provide benefits for genomic prediction, particularly in across-population prediction scenarios.

### Comparing prediction accuracies of high and low density marker panels

In the present study, prediction accuracies for CBSD3s and CBSDRs were 8% and 18% higher for high-density (WGS-imputed) markers than low-density (GBS) markers from single kernel G-BLUP model for the optimized training population of size of 200 clones (Table S11). Several studies have demonstrated increased prediction accuracies as a function of increase marker density (Peixoto *et al.*, 2016; Wang *et al.*, 2017). In a recent study, using NaCRRI training population, prediction for CBSD-related traits, in a single kernel G-BLUP model was not improved by whole-genome imputation (Lozano *et al.*, 2017). On the other hand, in a simulation study for across population genomic prediction in dairy cattle, De Roos *et al.*, (2009) reported higher prediction accuracies, similar to the improvement we observed for CBSD3s and CBSDRs, when more markers were included in the model. The study concluded that the reliability of genomic predictions across populations is determined by the consistency of marker–QTL allelic phase between the populations. The more diverged the populations are, the denser the markers must be to ensure preservation of marker–QTL phase across the populations. Increased prediction accuracies for CBSD3s and CBSDRs in this study, could therefore be a result of whole genome sequence imputed markers more reliably capturing the correct marker-CBSD QTL phase across the two populations. Since the only additional cost incurred in generating WGS-imputed markers is computational time, we believe that imputing the GBS markers to higher-density would benefit even poorly resourced breeding programs.

### Conclusion

We have presented the first empirical validation of genomic prediction for cassava brown streak disease across populations. Based on our results, training population optimization provided a benefit of increased prediction accuracies over random subset and full set of training population for foliar cassava brown streak disease. More importantly, inclusion of prior CBSD QTL information in our genomic prediction models reasonably increased foliar CBSD prediction increased for W. African clones. Furthermore, whole genome sequence imputed markers increased prediction accuracies for CBSD3s and CBSDRs. Future efforts to better predict CBSD resistance in W. Africa clones could focus initially on testing progeny from W. African germplasm, and later use the progeny evaluation data to train CBSD prediction models in W. African. Lastly, further research should target a much larger number of W. African test clones than we used in the current study.
